# Rapid luminescence-based screening method for SARS- CoV-2 inhibitors discovery

**DOI:** 10.1016/j.slasd.2025.100211

**Published:** 2025-01-15

**Authors:** Abdeldjalil Madani, Nadine Alvarez, Steven Park, Madhuvika Murugan, David S. Perlin

**Affiliations:** Center for Discovery and Innovation, Hackensack Meridian Health, 111 Ideation Way. Nutley, New Jersey 07110, United States

**Keywords:** Drug screening, HTS, Viruses, Antivirals, Nano-luciferase, Bsl-3 viruses, SARS-CoV-2, MMV global health priority box

## Abstract

The COVID-19 pandemic has emphasized the necessity for rapid and adaptable drug screening platforms against live pathogenic viruses that require high levels of biosafety containment. Conventional antiviral testing is time-consuming and labor-intensive. Here, we outline the design and validation of a semi-automated drug-screening platform for SARS-CoV-2 that utilizes multiple liquid handlers, a stable A549 cell line expressing ACE2 and TMPRSS2 receptors, and a recombinant SARS-CoV-2 strain harboring the nano-luciferase gene. This platform allows for accelerated low-, mid-, and high-throughput screenings by bypassing the virus inactivation and the staining steps compared to assays utilizing fluorescent reporter viruses or immunofluorescence. First, we demonstrated that the luminescence signal obtained at 24 h post-infection is robust and can be used as a surrogate for fluorescent reporter viruses and immunofluorescence assays that require 48 h incubation post infection. We confirmed the susceptibility of the reporter virus to a panel of reference drugs and validated the luminescence signal in 96- and 384-well plates in accordance with NIH criteria for high-throughput screening. The validation assays showed reproducible results, robust Z factor of ≥0.5, and a coefficient of variation of <20% achieved in both 96 and 384-well plate formats. Lastly, we assessed the assay’s performance by screening 240 compounds from the MMV Global Health Library, using the 384-well plate format and remdesivir as a control compound. The single point screening resulted in the identification of 48 hits that inhibited more than 50% of the viral growth. We selected the 15 most active compounds to evaluate their inhibitory concentration and their cytotoxicity, which resulted in the confirmation of the 3 most potent and least toxic compounds that were never reported as antivirals. These results confirm that our platform can be reliably employed for rapid drug screening against SARS-CoV-2 and can be easily adapted to other nano-luciferase reporter viruses.

## 1. Introduction

The novel severe acute respiratory syndrome coronavirus 2 (SARS-CoV-2) has led to a worldwide health crisis, including more than 775 million cases and over 7 million fatalities between 2019 and 2024 [1,2]. Although the COVID-19 health emergency has ended, SARS-CoV-2 infections continue to be reported, especially resulting in hospitalization of immunocompromised patients [3]. The ongoing emergence of new threats, such as H5N1/H7N9 avian influenza, Monkeypox and Nipah viruses, underscores the need for vigilance and preparedness [4–6]. To mitigate the risk of these and other potential pandemics, it is crucial to develop adaptable high-throughput screening (HTS) assays that can facilitate the rapid repurposing or discovery of effective drugs. By developing versatile tools, robust adaptable protocols and high-throughput drug screening platforms, we can better position ourselves to rapidly respond to emerging infectious diseases.

In response to the SARS-CoV-2 pandemic, several vaccine development programs and drug screening platforms emerged globally, resulting in the rapid discovery of mRNA vaccines and three FDA-approved drugs [7]. The mRNA vaccines have proven their effectiveness against the SARS-CoV-2 Wuhan strain and the variants of concern. However, the new Omicron subvariants evade the humoral immunity induced by mRNA vaccines, and it has been shown that the 2023–2024 updated COVID-19 vaccine provides only limited protection against the JN.1 lineage of SARS-CoV-2, necessitating the update of the vaccines as these sub-variants continue to evolve [8,9]. Although vaccines reduce severe outcomes from most of the population, new treatment options against SARS-CoV-2 are needed for vulnerable patients that cannot be vaccinated and/or have weakened immune status, as well as for new outbreaks.

The US Centers for Disease Control and Prevention (CDC) currently recommends only three drugs for the treatment of COVID-19, each with notable limitations. Remdesivir (VEKLURY^®^) requires intravenous administration; ritonavir-boosted nirmatrelvir (PAXLOVID^™^) is associated with drug-drug interactions; and molnupiravir (LAGEVRIO^™^) is contraindicated in pregnant patients [1]. The restricted number of available treatments and their associated drawbacks emphasize the urgent need for the development of safer and more effective therapeutics against SARS-CoV-2.

Since the onset of the pandemic, several screening platforms have been developed to evaluate compounds against SARS-CoV-2, including those that (i) screen against the main targets of SARS-CoV-2 [10–14], (ii) use SARS pseudo-typed particles for drug efficacy screening [15–16], or (iii) test the binding of drugs to the spike protein [17]. These platforms provide rapid results and do not require high-containment environments; however, they primarily offer predictive data that must be validated through antiviral activity testing against live viruses. Currently, in most countries including the United States, Canada, the European Union members, live SARS-CoV-2 assays can only be performed in biosafety level 3 (BSL-3) laboratories [18], underscoring the need for the development of more efficient workflows that can facilitate rapid antiviral screenings while addressing the constraints of BSL-3 containment.

Several live-virus assays rely on the phenotypic antiviral efficacy assay or cytopathic effect reduction (CPE) [19]; however, these assays are time consuming especially when coupled with image acquisition and processing. Alternative assays use fluorescent reporter viruses [20] or immunofluorescence techniques, which are labor-intensive requiring multiple staining steps. Recently a luciferase reporter version of WA1/2020 SARS-CoV-2 was engineered [21] and used in HTS settings [22,23]. The published methods using the SARS-CoV-2 nLuc require 48 h or 72 h of incubation post-infection or are typically limited to 96-well plate format. Here, we have developed and validated a more efficient drug screening platform that reduces incubation time to just 24 h post-infection. This platform supports low-, mid-, and high-throughput screening (LTS, MTS and HTS respectively) in both 96- and 384-well plates within 48 h. The platform employs the icSARS-CoV-2-nLuc system, a stable and highly permissive A549 cell line expressing ACE2 and TMPRSS2 [24] and utilizes several automated liquid handlers to streamline the process.

The first step in the development of the platform was the validation of the automatic liquid dispensing for both cell seeding, the viral infection and the addition of the reagent. The second step was the comparison of the luminescence readout to the immunofluorescence signal measuring the nucleocapsid proteins and the susceptibility of the reporter virus was assessed against a panel of commercially available reference drugs. The luminescence signal was assessed in 96- and 384-well plate formats to cover LTS, MTS and HTS and validated following the NIH recommendations for HTS [25]. Finally, we validated the method by screening the MMV Global Health Priority Box against icSARS-CoV-2-nLuc in 384-well plate format.

The advantages of the protocol are: (i) it is rapid and requires less manual handling; (ii) the assay is entirely performed in BSL-3 laboratory and does not require inactivation, staining, or imaging steps, (iii) it can be scaled up/down depending on the throughput (LTS, MTS, and HTS) (iv) several techniques outlined in this paper have the potential to enhance various assays that require the use of high-throughput liquid handlers. The validation approach described could be applied to and beyond other nano-luciferase reporter viruses as well. The limitations of this assay include: [1] the availability of appropriate instruments in the BSL-3 laboratory, and [2] the necessity for personnel training to work under BSL-3 conditions, and [3] the cost of the reagent for the nano-luciferase activity.

## 2. Materials

### 2.1. Biological material

- Vero E6 cell line expressing the transmembrane serine protease TMPRSS2 (Vero E6/TMPRSS2) was obtained from JCRB CELL BANK (Cat #JCRB1819).
- ACE2plusC3-A549/ACE2/TMPRSS2 cell line (C3) was kindly provided by Dr. Ching-Wen Chang.
- SARS-related Coronavirus 2 Isolate USA-WA1/2020 recombinant infectious Clone with Nano-luciferase Gene icSARS-CoV-2-nLuc was obtained from BEI Resources (#NR-54,003).

**Important:** icSARS-CoV-2-nLuc is a BSL-3 pathogen and any assay with this virus should be performed under BSL3-conditions and safety considerations.

### 2.2. Equipment

- BioTek EL406 Washer Dispenser (Agilent BioTek #406PUB1SN)
- Tecan D300e digital dispenser (Tecan #30,100,152)
- Agilent BioTek Cytation C10 Confocal Imaging Reader (BioTek #C10MPHC2)
- Tecan Infinite 200 (TECAN #1,705,007,031)
- I.DOT Liquid Handler (DISPENDIX #16,110,022,085)
- Integra VIAFLO 96 automated pipetting system (INTEGRA #6001)
- Hamilton MICROLAB STARlet Liquid Handler (HAMILTON #B998)
- Refrigerated centrifuge (Beckman coulter Allegra #X12-R)
- LUNA-II^™^ Automated Cell Counter (Logos biosystem # L40002)
- Inverted microscope (EVOS XL core AMEX1200)
- 6-well Clear TC-treated Multiple Well Plates, Individually Wrapped, Sterile (Corning #3516)
- 96-Well White Polystyrene Microplate (Corning^™^ #3903)
- 384-Well, Cell Culture-Treated, Flat-Bottom, Low Flange white Microplate (Corning^™^ #3765)
- 384-Well, Cell Culture-Treated, Flat-Bottom, Low Flange black Microplate (Corning^™^ #3764)
- S.100 Plate (DISPENDIX #16,110,021,802)
- T175 cell culture flask (Nunc^™^ #159,910)
- T8+ Dispensehead Cassettes (TECAN #30,097,370)
- D4+ Dispensehead Cassettes (TECAN #30,097,371)
- 200 mL Black Reagent Reservoir, Self-Standing with Lid (Hamilton #56,695–02)
- 300 ml Polystyrene Reservoir (Integra #6327)
- 300 ml Polystyrene Reservoir (Integra #6327)
- 125 μl standard tips (Integra #6465)
- 50 μL CO-RE^®^ II Tips (Hamilton #235,829)
- 300 μL CO-RE^®^ II Tips (Hamilton #235,830)
- BreatheEasy plate covers (Sigma-Aldrich #Z380059)
- Nalgene^™^ Rapid-Flow^™^ Sterile Disposable Filter Units with PES, CN, SFCA or Nylon Membranes (Thermo Scientific^™^ #567–0020)
- LUNA^™^ Cell Counting Slides (Logos biosystem # L12001)
- 37 °C water bath (Precision #51,221,050)
- 37 °C CO2 incubator (Panasonic #MCO-170AICUVH)
- Multichannel pipette 1–10 (Eppendorf^™^ #2,231,300,043)
- Multichannel pipette 10–100 (Eppendorf^™^ #2,231,300,045)
- Multichannel pipette 1–10 μL (Thermo Scientific^™^ # 4,662,000)
- Multichannel pipette 5–50 μL (Thermo Scientific^™^ # 4,662,010)
- 50 ml conical tubes (SARSTEDT #62–547–100)
- 5 mL Serological pipettes (Fisherbrand^™^ #13–676–10H)
- 10 mL Serological pipettes (Fisherbrand^™^ #13–678–11E)
- 25 mL Serological pipettes (Fisherbrand^™^ # 13–678–11)
- 50 mL Serological pipettes (Fisherbrand^™^ #13–678–11F)

**Alternatives:** The liquid handlers selected for the validation of this protocol are user friendly and BSL-3 friendly. Other liquid handlers might be used for the dispensing.

### 2.3. Reagents

- DMEM (GIBCO #10,569,010)
- FBS, heat inactivated (GIBCO #10,082,147)
- Antibiotic-Antimycotic (100X) (Gibco #15,240,062)
- 2X DMEM (Sigma-Aldrich #SLM202)
- PBS (GIBCO # 10,010,049)
- Dimethyl sulfoxide (Sigma #D8418)
- Formal Fixx (Epredia^™^ #9,990,244)
- Hoechst (Thermo Scientific^™^ #62,249)
- Agarose (Sigma-Aldrich #A9539)
- TrypLE^™^ Express Enzyme 1X, no phenol red (Gibco #12,604,039)
- Remdesivir (REM) GS-5734 (Selleckchem #S8932)
- Nirmatrelvir (PF-07,321,332) (AOBIOUS #AOB14800)
- Molnupiravir (EIDD-2801, MK-4482) (MCE HY-135,853)
- Ritonavir (ABT 538, RTV) (MCE HY-90,001)
- Narlaprelvir (SCH 900,518) (MCE HY-10,300)
- Boceprevir EBP 520, SCH-503,034 (MCE HY-10,237)
- Non-fat milk (RPI #M17200500.0)
- Nano-Glo^®^ Luciferase Assay System (Promega # N1130)
- Purified anti-SARS-CoV-2 Nucleocapsid Antibody (BioLegend #940,902)
- Goat anti-Mouse IgG (*H* + *L*) Cross-Adsorbed Secondary Antibody, Alexa Fluor^™^ 488 (Invitrogen #A11126)

### 2.4. Preparation of medium and solutions

- Complete DMEM (DMEMc) was prepared using DMEM supplemented with 10 % FBS, 1 % Antibiotic-Antimycotic and filtered through a 0.22 μm filter system.
- The agarose overlay was prepared by mixing 1:1 vol of 1.6 % melted agarose in sterile water and 2X DMEM supplemented with 10 % FBS and 1 % Antibiotic-Antimycotic. The overlay was prepared the day of the infection in a 50 mL conical tube and kept at 45–50 °C.
- Blocking buffer was prepared with PBS 1x and 3 % non-fat milk.

### 2.5. MMV global health priority box

The library was kindly provided by Medicine Malaria Venture (MMV) to accelerate the development of new drugs against SARS-CoV-2. This box holds a collection of 240 compounds with confirmed activity against different pathogens and vectors. Further details regarding the Global Health Priority Box can be found at the website for Medicines for Malaria Venture (mmv.org).

### 2.6. Software

- Microsoft Office Excel 2021
- GraphPad prism (version 10.0.0)
- Venus software V3.1

#### 2.6.1. Procedure

The procedure describes a series of steps followed to perform single point screening (SPS) and dose-response experiments against icSARS-CoV-2-nLuc.

### 2.7. Cell culture

**Timing:** 1 h (starting a new culture); 3 days of incubation, and 1 to 2 h on day 4 (cell seeding).

This step was performed in a BSL-2 laboratory. In this study we used VeroE6/TMPRSS2 for virus purification and propagation, and the C3 cell line for the validation and the drug screening. Note that the growth conditions was adjusted for each cell line.

#### 2.7.1. Starting a new cell culture

- The DMEMc was pre-warmed in a water bath at 37 °C.
- A stock of 1 × 10^6 cells was quickly thawed at 37 °C; then the cells were transferred aseptically into a 50 mL conical tube containing 5 mL of pre-warmed DMEMc.
- The cells were centrifuged for 5 min at 1500 RPM.
- The supernatant was removed, and the cell pellet was resuspended gently in 30 mL of pre-warmed DMEMc.
- The cells were transferred to a T175 vessel and incubated at 37 °C for 3–4 days.

**Critical:** The following day the cells were checked under a microscope for adherence (healthy cells should be attached). On the subsequent days, the cells were monitored daily for growth, morphology changes and confluency evaluation.

#### 2.7.2. Cell detachment and counting

- Once the cells reached 80% to 90% confluency (after 3 days), the medium was removed from the flask without disturbing the cell monolayer.
- 8 mL of TrypLE were added and the flask was incubated for 10 min at 37 °C to allow the cell detachment.
- Once the cells were detached, 10 mL of pre-warmed DMEMc were added to the flask to inactivate the trypsinization step.
- The cells were recovered by washing gently the flask surface, then the cell suspension was transferred to a 50 mL conical tube.
- The cell suspension was centrifuged for 5 min at 1500 RPM.
- The supernatant was removed, and the cell pellet was resuspended in 10 mL of pre-warmed DMEMc
- The cells were counted by transferring 10 μL of the cell suspension into a Luna cell Counting slide and then by using the Luna II automated cell counter.
- The cells were then used for seeding microplates (see subchapter 1.3) or starting a new culture by adding 1 × 10^6 cells into a T175 containing 30mL of pre-warmed DMEMc.

**Important:** The cell lines can be maintained in T175 flasks for up to 15 passages before starting a new culture using a new stock.

#### 2.7.3. Cell seeding in microplates

- We used three different types of microplates in this study, different cell suspensions were prepared according to each plate type in 50 mL conical tubes as follow; [1] 6 well plates for virus purification were seeded with 1 million cell per well [2] 96 well-plates for the signal validation were seeded with 1 × 10^4 cells per well and [3] 384-well plates were seeded with 3 × 10^3 cells per well.
  - For the 6-well plate seeding, we prepared a cell suspension of 13 mL using DMEMc containing 5 × 10^5 cell per mL and seeded 2 ml per well using a serological pipette.
  - For the 96-well plates, we prepared a cell suspension of5 mL using DMEMc containing 2 × 10^5 cells per mL and seeded the plates with 50 μL per well using the EL406.
  - For the 384 well plates, we prepared a cell suspension of 7.7 mL using DMEMc containing 1.5 × 10^5 cells per mL and seeded the plates with 20 μL per well using the EL406.

**CRITICAL:** The volumes prepared for each plate should be adjusted to cover the dead volumes associated with automatic liquid handlers. The EL406 tubing needs to be primed with 600 μL per tube and requires at least 4.8 mL of extra cell suspension. To ensure an equal cell dispensing in the wells the cell suspension should be mixed well before the dispensing of each plate.

**Alternatives:** 96-well plates can be dispensed manually using a multichannel pipette or automatic dispensing can be done using other liquid handlers like the Integra Viaflo 96.

#### 2.7.4. Drug dispensing

**Timing:** 30 min to 1 h of processing, 2 h of incubation.

- 10 mM stocks of the drugs to be tested were prepared in DMSO a day prior to the dispensing and kept at −20 °C.
- On the day of the dispensing, the drugs were thawed at room temperature.
- The Tecan D300 was used to dispense drugs for the signal validation experiments and dose response experiments, and the I-Dot was used for both signal validation and the screening of the MMV library.
- In this study we dispensed the drugs as follow:
  - For the HTS signal validation experiment, REM was used at two concentrations, [1] 0.1 μM for the mid signal; [2] 1 μM for the low signal. In addition, DMSO was used for the high signal.
  - For the single point screening evaluation of the MMV library, the compounds, and the controls REM were dispensed at 10 μM final concentration.
  - For the IC50 determination, different 9-point concentration ranges were used as described in the supplementary material (S1, S2 and S5).
  - For all the experiments, the total volume of DMSO per well did not exceed 0.5%.
  - A normalization to the highest volume of DMSO was applied to the wells.
  - After drug dispensing, the plates were incubated for 2 h at 37 °C then transferred to the BSL-3 laboratory for the infection steps.

**Note:** All the plate layouts used in this study are shown in supplemental material S4 and S5.

#### 2.7.5. Quality control of the cell dispensing

First, the volume dispensed by the EL-406 or the Viaflo 96 were checked manually in both 96- and 384-well plates using multichannel pipettes. Then, the number of cells dispensed in each well was evaluated 24 h after seeding by using Hoechst staining and counting the cells with the Cytation C10 plate reader.

### 2.8. Plaque purification of icSARS-CoV-2-nLuc

**Timing:** Four days.

This step was performed in a BSL-3 setting. The goal of the purification step was to obtain a clone that provides high infectivity and strong luminescence signal. The purified clone was used in step 2 to prepare a large stock of homogeneous viral population.

**Important:** Vero E6/TMPRSS2 cell line was used for the virus purification due to the large size of the plaques obtained with this cell line after three days of infection. The size of the plaques facilitated the PFU count and the recovery of the purified virus.

- Twenty-four hours prior to the virus purification, a 6 well plate was seeded as described before and incubated at 37 °C and 5 % CO_2_ overnight.
- On the day of the infection, the 6-well plate was checked under the microscope for cell attachment and confluency before being transferred to the BSL-3 laboratory.
- The agarose was prepared as described in the material section the same day of the experiment and kept in a water bath at 45 °C then transferred to the BSL-3 laboratory and kept at 45 °C.
- In the BSL-3, a vial of the virus stock was thawed at room temperature.
- Five serial dilutions from 10 ^to 1^ to 10^−5^ were prepared in 5 × 1.5 mL eppendorf tubes by transferring 20 μL from the virus stock to the first tube containing 180 μL of DMEMc and vortexing, then by repeating the same step for the next tubes.
- The medium from the 6-well plate was removed, then the cells were infected by adding 200 μL of the virus dilution to the corresponding well.
- The plate was incubated for 1 h at 37 °C and 5 % CO2, and was gently rocked by hand every 15 min to make sure the inoculum was evenly distributed.
- After the infection, the wells were washed once with PBS 1X, and 2.5 mL of the agarose overlay was added to each well. The plate was incubated at room temperature for 5 min for the agarose solidification, followed by 3 days incubation at 37 °C and 5 % CO_2_.
- On day three, of the five serial dilutions, the dilution showing the lowest number of plaques that were also clearly separate was selected for the next steps.
- The agarose on top of the plaques was stabbed with a 200 μL micropipette and regular filter tips to aspirate the liquid (15 to 20 μL of suspension was recovered from each plaque).
- The recovered suspensions were transferred to tubes containing 500 μL of DMEMc and vortexed for few seconds.

**Important:** The viral suspension recovered can be used on the same day for the virus propagation or stored at −80 °C for later use.

### 2.9. Virus propagation

**Timing:** Four days.

- The virus was propagated by infecting a semi-confluent T175 flask containing Vero E6/TMPRSS2 cells in 30 mL DMEMc with 100 μL of the purified virus suspension. The flask was incubated for 3 days at 37 °C and 5 % CO_2_.
- Three days later, the CPE was evaluated under microscope, and the supernatant was collected in a 50 mL falcon tube.
- The supernatant was centrifuged for 5 min at 1500 RPM and 500 μL single use stocks were prepared and stored at −80 °C.
- The titer of the stocks was determined by performing a plaque assay using Vero E6/TMPRSS2 cells. Briefly, the supernatant harvested was serially diluted and inoculated on a cell monolayer in a 6 well plate, then overlaid with 2x MEM & 2.5% CMC in 1:1 ratio. Plaques were visualized at 72h after performing crystal violet staining.

### 2.10. Drug screening protocol

In this step, the activity of subject compounds is assessed against icSARS-CoV-2-nLuc in a single point concentration or using an appropriate concentration range in a dose response experiment.

#### 2.10.1. Viral infection

**Timing:** 1–2 h for the plate infection, plus 24 h of incubation.

- A vial of the purified icSARS-CoV-2-nLuc was thawed at room temperature.
- The virus suspension was prepared in 50 mL conical tubes containing DMEMc to achieve MOIs of 0.005, 0.01, 0.02, 0.05 and mixed by inverting the tube several times and vortexing.
- The plates were infected using either Viaflo 96 or the Hamilton STARlet by dispensing 20 μL of the virus suspension per well in the 384-well plates or 50 μL per well in the 96-well plates.
- The infected plates were incubated for 24 h at 37 °C and 5 % CO_2_.

**Note:** The Viaflo 96 is more convenient for low- and mid-throughput screening especially when infecting less than 10 plates while the Hamilton STARlet is more convenient for HTS experiments with a fully automatic liquid dispensing in up to 10 plates at a time.

#### 2.10.2. Plate reading

**Timing:** 1 h

- The Nano-glo reagent was prepared by mixing 200μL of the substrate to 10 mL of the buffer.
- The Nano-glo reagent was then added to the plates by dispensing 100 μL per well in the 96-well plates or 40 μL per well in the 384-well plates using either the Viaflo 96 or the Hamilton STARlet.
- The plates were covered with Breatheasy plate covers and incubated for 5 min at room temperature.
- The plate surface was decontaminated before being transferred into the plate reader.
- The luminescence signal was detected using the Tecan Infinite 200, an integration time of 300 ms and an automatic attenuation.

### 2.11. Troubleshooting

The issues faced during the optimization of the process and their solutions are described in the table below.

**Table:** 

Cell seeding issues	

Issue	Solutions
Cells exhibiting signs of distress or not confluent	Use fresh media and reduce time outside the CO2 incubator
Malfunction of the liquid handler	Respecting the dead volumes Checking the heads for clogs (for EL-406) Checking the consistency of the volume dispensed Tapping gently the plates after dispensing to Remove air bubbles from wells Flushing the tubing system after dispensing (for EL-406)
**Luminescence signal issues**	
**Potential issue/origin**	**Solution**
Cell seeding issues	**Control the quality of the cell culture and the seeding:** maintain healthy cells and validate the automatic dispensing before running the experiments
Liquid handler issues	**Maintain accurate dispensing:** Regularly check and maintain the accuracy of liquid handlers. For devices like the Integra Viaflo 96 without automatic liquid control, include a pause step to visually inspect the tips during dispensing.
Virus suspension	**Thoroughly Mix Virus Suspension**: Ensure the virus suspension is well-mixed by inverting conical tubes several times and vortexing.
Signal overlap from positive controls	**Optimize Experimental Conditions**: Determine the optimal multiplicity of infection (MOI) and incubation time to avoid exceeding the detection limit. To prevent signal bleeding into adjacent wells, cover the bottom of the plate with black tape
Assay volume variability	**Adjust Assay Volumes**: We noted that increasing assay volumes from 25μL to 40μL reduced the variability in the luminescence signal between replicates. However, since the nLuc reagent is costly and typically used in a 1:1 ratio, the cost of reagents needs to be carefully considered when designing the assay.

**Note:** In high-throughput screening (HTS), considerations are often made based on cost, reagent consumption, and physiological conditions. For single-point screening with rare or expensive chemical libraries, ensuring consistent data is crucial and justifies the reagent expenses to avoid repetition.

### 2.12. Data analysis

NIH guidelines were followed to run the assays and to calculate all the statistical parameters [22]. All data was analyzed using GraphPad Prism (version 10.0.0) software package. The outliers are marked with # in all the supplementary material. The data was normalized to the control DMSO (considered as 0 % inhibition), and to the non-infected cells (considered as 100 % inhibition). The dose-response curves were fitted and the IC_50_ values were determined using a non-linear regression analysis (curve fit), applying the equation for inhibitor versus response-variable slope (four parameters) and 95 % confidence interval.

### 2.13. Additional method used in this study: virus immunofluorescence assay

This extra step was used to correlate the luminescence signal to the immune-fluorescence signal to validate the use of icSARS-CoV-2-nLuc.

**Note:** For the immunofluorescence assay, all the dispensing and washing steps were performed using the EL406 washer dispenser. The tubing was primed before each step with 600 μL of the appropriate reagent and flushed after dispensing. Viral infection. The immunofluorescence assay requires 48 h of incubation.

#### 1.13.1. Plates fixation

- In the BSL-3, 40 μL of FormalFixx was added to the 384-well plates infected with icSARS-CoV-2-nLuc using the Viaflo 96 or the Hamilton STARlet.
- Then, the surface of the plates was decontaminated, and the plates were stored at 4 °C for 24 h
- The next day, the fixed plates were transferred to a BSL-2 laboratory for the staining.

**Note:** The addition of the FormalFixx is an approved protocol for inactivating SARS-CoV-2 for removal from BSL-3 containment at the Center for Discovery and Innovation.

#### 2.13.2. Plate staining using the EL406

- The plates were washed twice with 50 μL/well of PBS 1X.
- Twenty-five microliters of the blocking buffer were added to the wells then the plates were incubated for 1 h at 4 °C.
- The plates were washed twice with PBS 1X.
- Twenty-five microliters per well of Purified anti-SARS-CoV-2 Nucleocapsid Antibody (working solution of 1:10,000, prepared in PBS) were added and the plates were incubated at 4 °C overnight.
- The next day, the plates were washed twice with PBS 1X.
- Twenty-five microliters of goat anti-mouse IgG (*H* + *L*) Cross-Adsorbed Secondary Antibody, Alexa Fluor^™^ 488 (working solution of 1:20,000 prepared in PBS) were dispensed in each well then, the plates were left at room temperature for 1 h
- The plates were washed twice with PBS 1X.
- Twenty-five microliters per well of Hoechst (final concentration of 5 μg/mL) were added and the plates were incubated for 10 mins at room temperature.
- The bottom of the plates was wiped with 70 % ethanol and the plates were scanned with the Agilent Bio Tek Cytation C10 using DAPI and Texas red filters.

## 3. Results

### 3.1. Condition optimization and susceptibility testing of the icSARS-CoV-2-nLuc

**Fig. 1.**
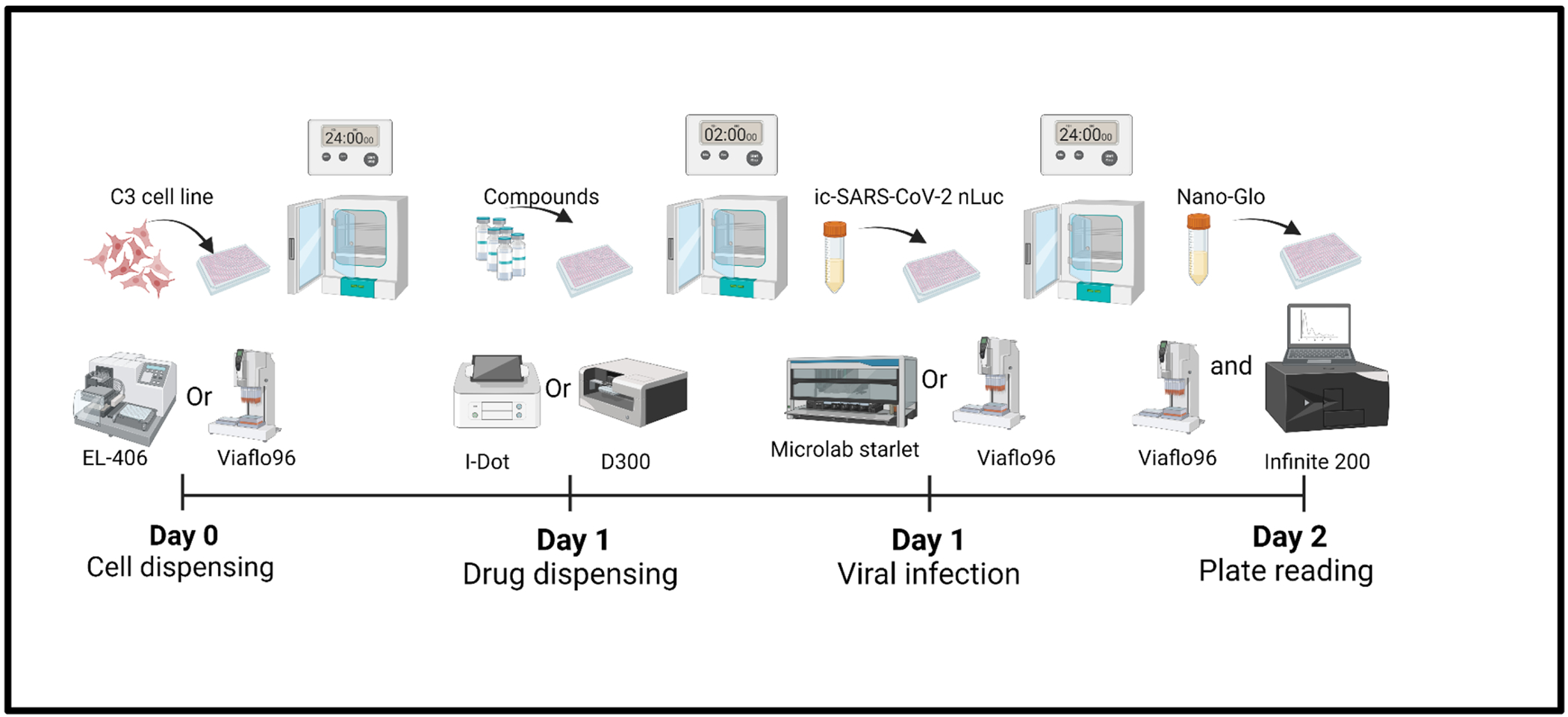
Schematic representation of the screening platform design. The overall process runs over 3 days and is divided into 4 major steps: **(1) cell dispensing:** processing time of 1 to 2 h for the cell detachment, cell counting and seeding with the EL-406 or the Integra-96. The cells are then incubated 24 h at 37 °C and 5% CO2 for attachment); **(2) drug dispensing:** processing time of 1 h using the Tecan D300e or the I-dot liquid Dispendix followed by 2 h of incubation; **(3) viral infection:** processing time of 1 to 2 h for the virus suspension preparation and the plate infection with the Viaflo-96 or the Hamilton Starlet, followed by 24 h of incubation for the virus growth at 37 °C and 5% CO2; **(4) plate reading:** processing time 1 h for the Nano-glo preparation and dispensing using the Viaflo-96 or the Hamilton Starlet, then the plate reading with the Tecan Infinite 200.

It has been demonstrated previously that the ic-SARS-CoV-2-nLuc virus performs effectively in LTS assays using 384 well plates format and an incubation of 72 h post infection [22]. To further expedite the process and increase the screening capacity, we designed a semi-automatic platform that can run LTS, MTS and HTS, in both 96- and 384-well plate formats (Fig. 1) and requires only 24 h of incubation post infection. This platform leverages various liquid handling systems available in our BSL-2 and BSL-3 laboratories, optimizing efficiency and throughput.

**Fig. 2.**
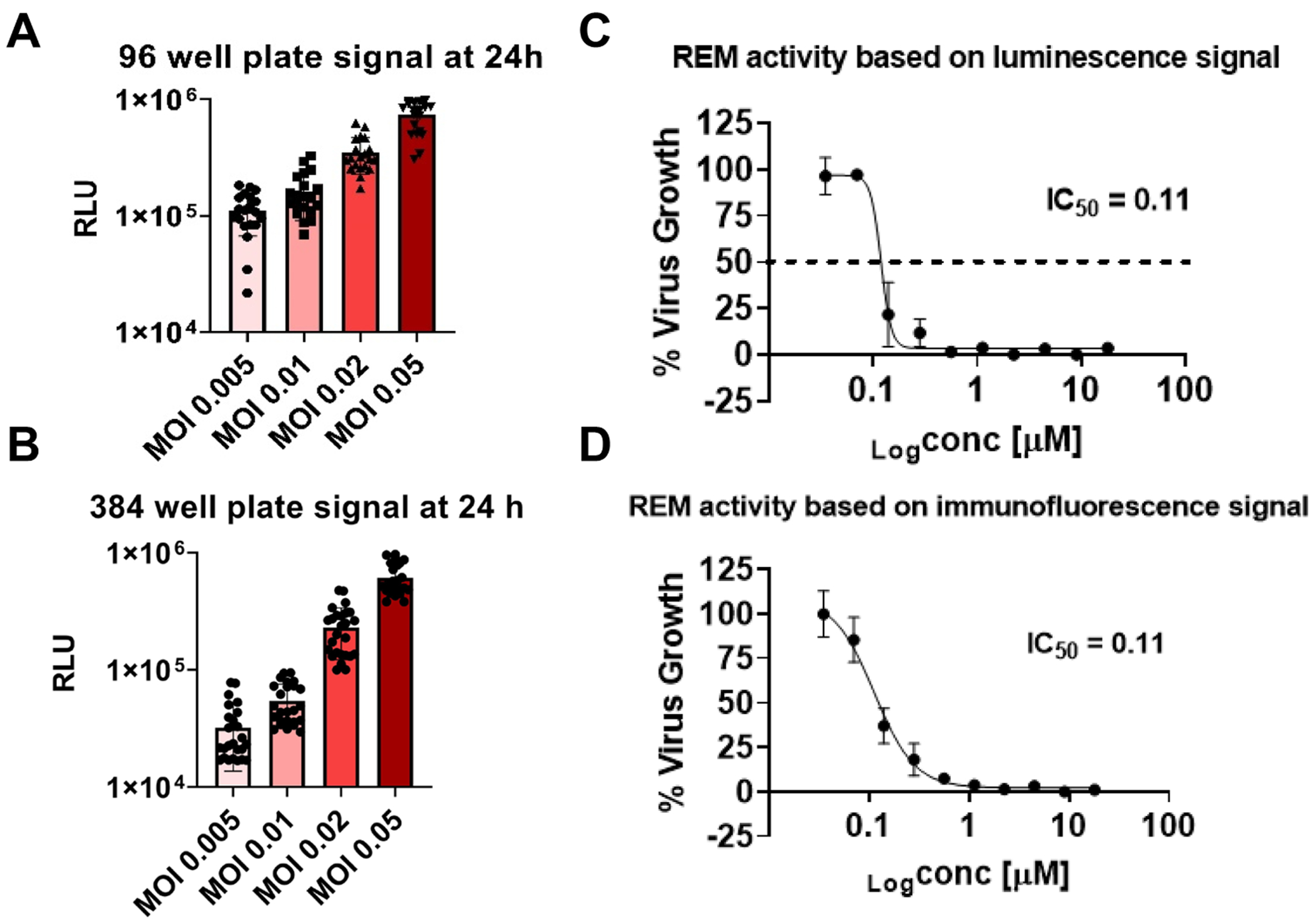
Signal optimization and validation and icSARS-CoV-2-nLuc susceptibility to remdesivir using luminescence and immunofluorescence. A) luminescence signals obtained at 24 h post-infection using different MOIs (0.005, 0.01, 0.02, 0.05) in 96 well plate format, using 20 well for each condition and 16 non infected wells. B) luminescence signals at 24 h post-infection using different MOIs (0.005, 0.01, 0.02, 0.05) in 384 well plate format using 24 well for each condition. C) IC_50_ curve of REM against icSARS-CoV-2-nLuc obtained at 24h post infection with luminescence data, a serial dilution of 2 fold was used and 3 technical replicates for each concentration from 18 μM to 0.035μM, the data shown are means and standard deviations for the three replicates. D) IC_50_ curve of REM against icSARS-CoV-2-nLuc obtained at 48h post infection with immunofluorescence data. A serial dilution of 2 fold was used with 3 replicates for each concentration from 18 μM to 0.035μM, the data shown are means and standard deviations for the three replicates obtained.

To validate our assay conditions, we assessed the signal of the purified icSARS-CoV-2-nLuc in the C3 cell line at 24 h post infection in the 96- and 384-well plate formats using different MOIs (0.005, 0.01, 0.02, 0.05). As expected, no significant CPE was observed at any of the MOIs used after 24 h of incubation (S7), and the luminescence signal obtained ranged from 5 × 10^4 to 1 × 10^6 correlating with the MOIs used (Fig. 2A and B). This result indicates that the luminescence-based assay is more sensitive and provides a robust signal at 24 h post infection compared to the assays relying either on fluorescent reporter viruses or Immunofluorescence which require longer incubation time at a higher MOI. Hence, the MOI of 0.05 and the incubation time of 24 h were selected as the optimal conditions for the following assays.

Furthermore, we tested the susceptibility of the icSARS-CoV-2-nLuc to REM in both luminescence-based assay and immunofluorescence assay. The IC_50_ obtained from both assays was 0.11 μM (Fig. 2C, D and S1), which is consistent with previously reported activity for REM against the SARS-CoV-2 WT strain [26], affirming both the robustness of the luminescence-based assay and the WT phenotype of the reporter virus.

**Fig. 3.**
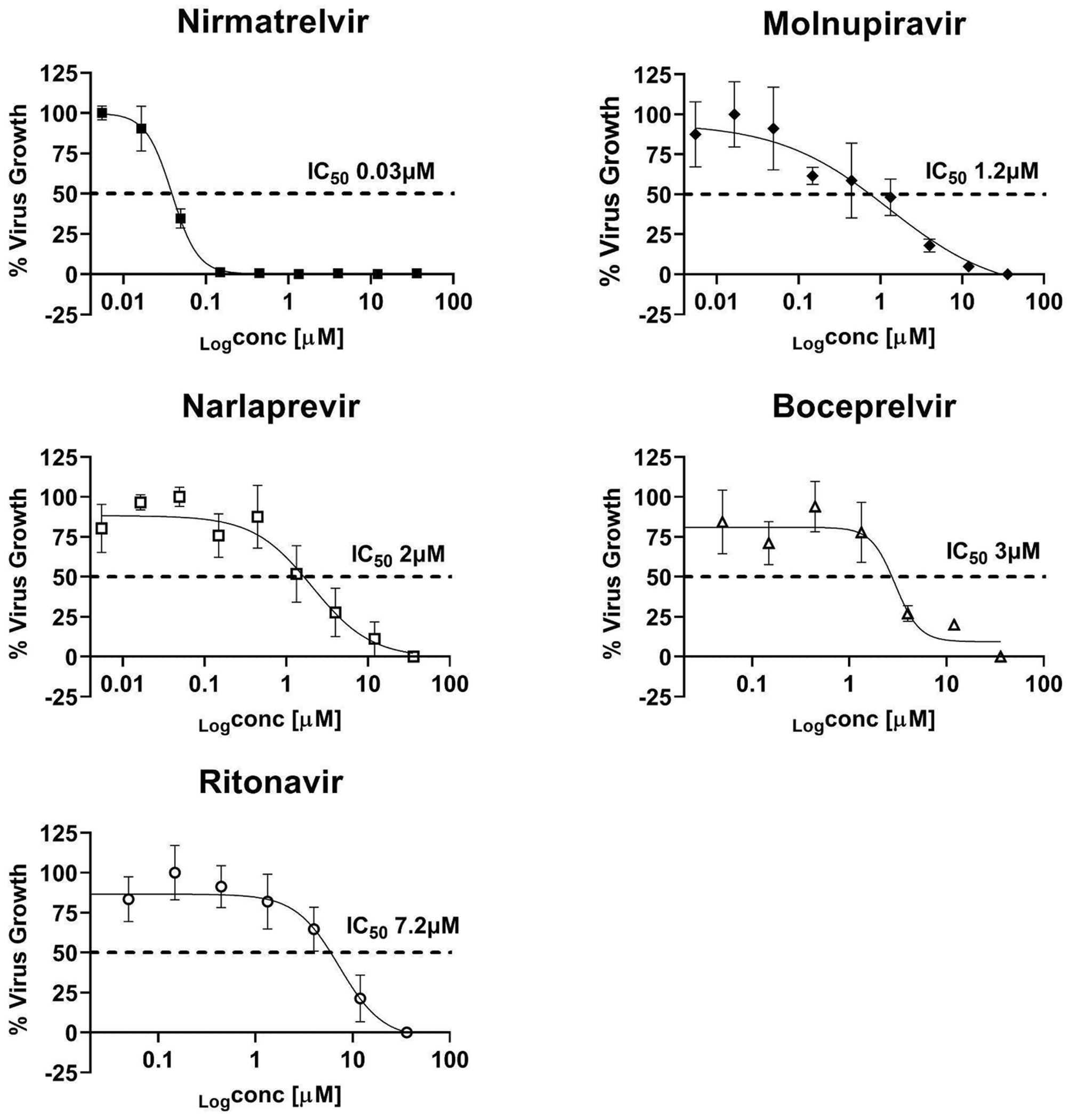
Susceptibility testing of the icSARS-CoV-2-nLuc to reference drugs using luminescence signal readout. The C3 cells were seeded in 384-well plate and treated 2 h before infection with nirmatrelvir, ritonavir, molnupiravir, boceprevir, and narlaprevir using a serial dilution of three-fold from 36 μM to 0.005 μM and 2 technical replicates for each concentration. The cells were infected with icSARS-CoV2-nLuc at MOI 0.05 and incubated for 24 h at 37 °C and 5% CO2. On the next day, the nLuc activity was measured and the data was analyzed using Graphpad prism software to obtain the IC_50_ curves. The graph represents the result of two independent experiments.

In addition, we evaluated the susceptibility of the icSARS-CoV-2-nLuc to a panel of reference drugs with established in vitro activity against the SARS-CoV-2 wildtype strain. The panel included nirmatrelvir, ritonavir, molnupiravir, boceprevir, and narlaprevir (Fig 3.) All the tested drugs showed IC_50s_ in the same range as their reported activity against the SARS-CoV-2 wildtype strain [22,27–29], demonstrating that the icSARS-CoV-2-nLuc can be used for drug activity testing. For this assay, the signal to background ratio (SB) and the Z’ factor were calculated to assess the quality using the high signal obtained using DMSO and the low signal using 1μM of REM, for the 96 well plates the SB was 9.8 (± 0.3) and the Z’ was 0.61 (±0.04) and for the 384 well plate format the SB was 9.1 (± 2.8) meeting the requirement for good quality data.

### 3.2. Signal validation for HTS experiments

**Fig. 4.**
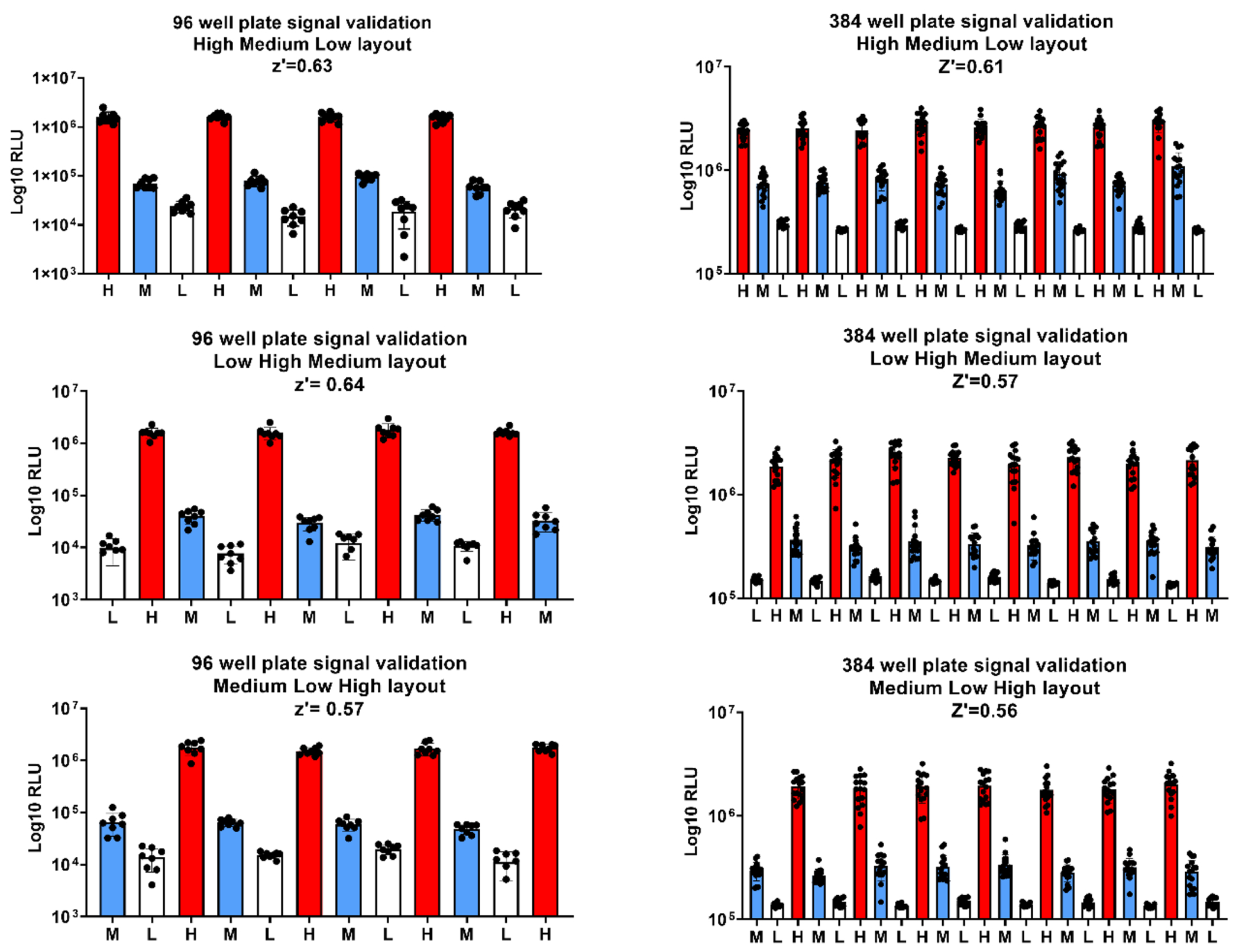
Luminescence signal validation for HTS assay in 96- and 384-well plates following the NIH guidelines. The C3 cells were seeded in 96 well plates at 10^4 cells per well or 384 at 3 × 10^3 cells per well and treated with REM or DMSO as shown in (sup mat 3) and incubated for 2 h prior the infection with icSARS-CoV-2-nLuc at MOI 0.05. After 24 h of incubation at 37 °C and 5% CO2, the nLuc activity was measured from the different plates and the data was plotted using Graphpad prism software. The data represents one of the three independent experiments. **A)** Signal validation in 96-well plates showing low signal (well with 1 μM of REM), mid-signal (wells with 100 nM of REM) and high signal (wells containing DMSO). **B)** Signal validation in 384-well plates using the same conditions described for the 96 well plates for high, mid, and low signal. The Z’ factors calculated for all the plates were above 0.5.

We validated the luminescence signal for HTS assays by following the NIH guidelines in 96- and 384-well plate formats [25]. We assessed the plate uniformity and the signal variability in three independent experiments using the standard interleaved format plates layout in 96- and 384-well plate format as described in the supplementary material S3 and S4. The DMSO was used to obtain the maximum signal of luminescence and the REM at 0.1 μM and 1 μM to obtain the mid and low signals, respectively. The signal was validated by running three independent assays, with three different plate layouts each time. The data was analyzed using the template provided with the NIH guidance where the mean (AVG), SD and CV (of the mean) for each of the three signals were calculated for all the plates (S3 and S4). The high-, mid- and low signals obtained were consistent between all the plates (Fig 4, S3 and S4). The Z’ factor for the plates used for signal validation ranged from 0.56 to 0.64, and the CV of each signal was less than 20 % in all the plates. These results met all the acceptance criteria of the NIH guidance for HTS signal validation, confirming that the luminescence-based assay can be used for HTS assays in 96- and 384-well plate format in a shorter time scale.

### 3.3. Screening of the mmv global health priority box

**Fig. 5.**
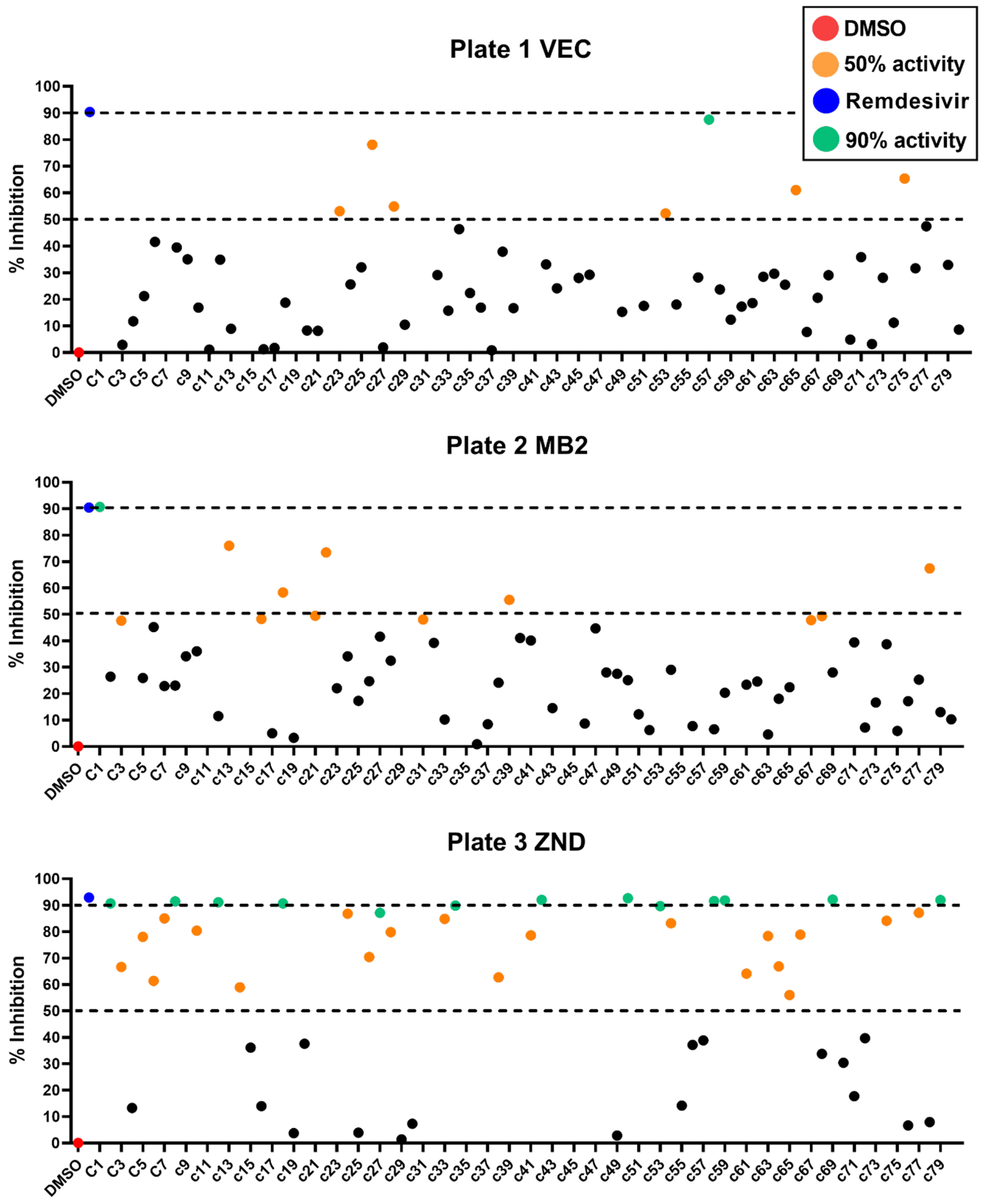
Scatter plots representing the single point screening of the MMV Global Health Priority box against icSARS-CoV-2-nLuc. A total of 240 compounds (80 from VEC plate, 80 from MB2 plate and 80 from ZND plate), were evaluated at 10 μM against icSARS-CoV-2-nLuc using MOI 0.05 in 384 well plate format. REM was used as a positive control of inhibition (blue square) and DMSO as a negative control (red square). The activity of the compounds is represented as: 0–49% inhibition (black dots), 50–89% inhibition (orange dots) and 90% or higher inhibition (green dots), the compounds that showed cytotoxicity (>50%) toward C3 cell line at 10 μM are represented by the brown dots. The graphs are representative of two independent assays and the represented activity of each compound is the average of the technical replicates. The compounds showing > 0% inhibition are not shown in the graph.

**Fig. 6.**
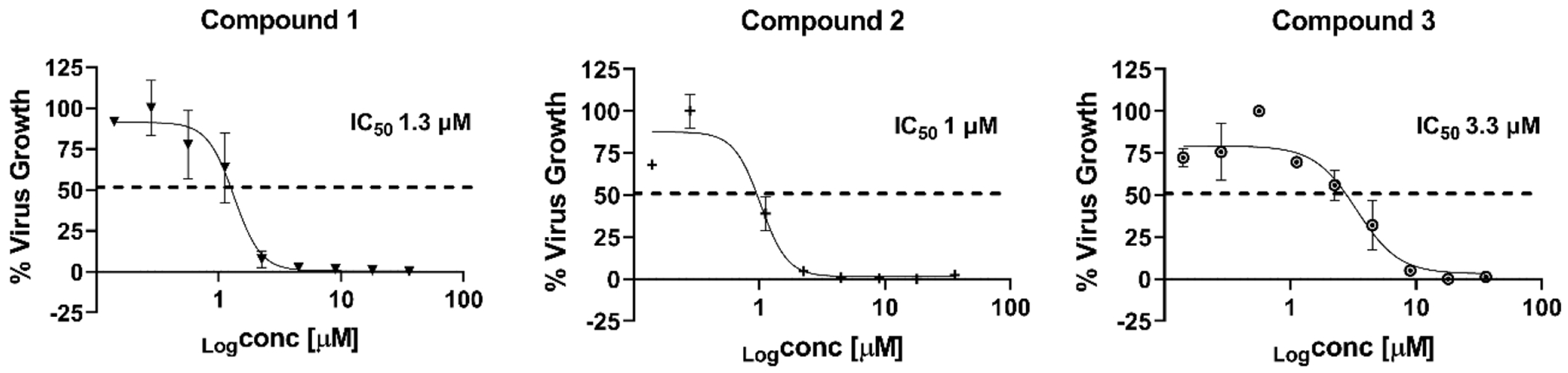
IC50 dose-response curves of three hits against icSARS-CoV-2-nLuc. This experiment was performed in 384 well plates using the C3 cell line. The attached cells were treated for 2 h with the selected hits from the SPS using two-fold dilution concentrations ranging from 36 μM to 0.018 μM. The cells were then infected with icSARS-CoV-2-nLuc at MOI 0.05 and incubated for 24 h at 37 °C and 5% CO2. The nLuc activity was measured and the IC_50s_ values were determined using Graphpad prism software. These data represent the result obtained from two independent assays.

To demonstrate the robustness of the screening platform, a 240-compound library from the MMV Global Health Priority Box was screened at a final concentration of 10 μM in the 384-well format. All the compounds from the library and the control REM were screened using quadruple technical replication (Fig 5, S5) in two independent biological replicates and the cytotoxicity of each compound was tested at the same concentration towards the C3 cell line. In the 384-well plates used for single point screening (Z’= 0.43–0.46), 48 compounds that demonstrated reduction in the luminescence signal with high correlation between replicates were considered. Among the 48 compounds, 18 showed toxicity toward the C3 cell line at 10 μM, 16 inhibited between 50 % and 80 % of the virus growth and 14 compounds inhibited 90% or more of the virus growth similar to REM at the same concentration. The rate of false positive hits was 2.5% between the two biological replicates. We selected fifteen compounds that showed activity in both assays for IC_50_ determination (S5) and cytotoxicity testing (S6). Most of the compounds showed moderate activity against the ic-SARS-CoV-2-nLuc with IC_50_ values between 4 and 12 μM. Three compounds showed high viral inhibition, with IC_50_ values between 1 μM and 3 μM (Fig. 6). The 3 compounds have CC_50_ equal to- or higher than 36 μM. Based on available information, these three compounds have not been tested against SARS-CoV-2 nor have any reports of antiviral activity, and therefore warrant further investigation.

### 3.4. Advantages and limitations of the assay

There are many advantages of using this assay: (i) using the highly permissive cell lines A549 expressing both ACE2 and TMPRSS2 receptors resulted in a stronger luminescence signal at 24 h (ii) the short incubation time after infection of 24 h allows multiple experiments to be run during the week, which gives a huge advantage compared to other assays relying on CPE or using fluorescent reporter viruses that require 48 to 72 h of incubation post infection. (iii) Less steps, handling, and easy readout; this assay does not require fixation of the plates at the end of the experiment or staining steps that are usually performed when using BSL-3 wild-type viruses. (iv) Beyond the use for small molecule library screening against icSARS-CoV-2-nLuc, this assay platform can be adapted and optimized for other therapeutic development including screening biologics such as neutralizing antibodies and synthetic peptides and other viruses. Compared to other throughputs using single-target assays, multitarget assays, or phenotypic antiviral efficacy assays, using nLuc reporter viruses allows for the rapid screening of compounds against live viruses and covers all the inhibitory actions that an antiviral can have. In fact, this study showed the robustness of the assay by the discovery of novel antivirals with unknown mechanisms of action that are currently under investigation. The limitations of this assay are: (i) the liquid handlers need to be maintained and the quality of the dispensing needs to be monitored to ensure optimal assay conditions; (ii) minimizing the assay volumes to increase the screening capacity can increase the variability of the signal, we noticed that using the 384-well plate format, the z scores obtained were acceptable but it shows that the conditions can be further optimized. (iii) to respect the NIH guidelines for HTS conditions the minimal number of replicate per plate for 96 well plate format is 2 and for the 384 well plate format is 4 in our conditions, which limits the number of compounds screened per plate; (iv) the use of Nano Glo reagent to read the luminescence signal may increase the cost of screening large compound libraries; (v) some compounds interfere with the luminescence signal and require other assays to be tested (vi) the training requirements and mandatory shutdowns for the BSL3 laboratory during maintenance pose additional challenges; and (vii) the availability of BSL-3 viruses with the nLuc reporter gene may be limited but this assay can easily be adapted to perform throughputs against other pandemic viruses and reporter viruses by modifying the assay conditions (cell line, media and incubation) and adding extra steps of fixation, staining and imaging.

## 4. Discussion

High-throughput screening (HTS) is an efficient methodology for identifying novel lead compounds in antiviral drug development. Since the onset of the COVID-19 pandemic, numerous studies have reported the development of novel HTS assays for the discovery of novel inhibitors and for the repurposing of FDA-approved drugs against SARS-CoV-2. Several HTS assays are single-target assays that rely on biochemical assays to assess the activity of libraries against the different SARS-CoV-2 proteins, like the main protease, the RNA-dependent RNA polymerases, the papain-like proteases, or the helicase [11,13,30]. Recent example of a single target HTS is the study measuring SARS-CoV-2 helicase activity in a 1536 well-plate format [30]. This assay facilitated the screening of 650 k compounds against the nsP13 helicase and the identification of several promising candidates for further optimization. Additional HTS assays targeting SARS-CoV-2 are designed to screen for entry inhibitors, either by interacting with the virus receptors, and an example is the reduction of endogenous TMPRSS2 expression [31] or by utilizing pseudotyped virus that incorporate the spike proteins of SARS-CoV-2 without the viral genome [16]. All the aforementioned HTS assays possess the advantage of being conducted in BSL-2 facilities, they are optimized for rapidity and efficacy and have the potential to lead to the discovery of a hit with a known mechanism of action. However, focusing on a single target is inherently limiting because (i) every hit necessitates confirmation in a cell-based assay against a live virus in a BSL-3 facility, and (ii) large libraries of small molecules can be prohibitively expensive, and screening them against different targets of the same virus using different assays might be labor intensive and time consuming.

The alternative to single-target assays is to use cell-based assay and a live virus to assess the activity of the test compounds. Most of these assays rely on measuring the prevention of the cytopathic effects (CPE) induced by the virus and are called CPE assays. These can be conducted via imaging or by measuring the ATP levels; however, they necessitate extended incubation periods of 72 h and substantial virus replication. The alternative to CPE assays is to measure the viral load directly through immunofluorescence, using reporter viruses or applying the AlphaLISA drug screening assay [11,32]. Applying CPE assays or measuring the viral load for HTS assay have demonstrated high robustness and reproducibility; nevertheless, when applied to a BSL3 virus, these assays present technical challenges for the execution and require additional handling for virus inactivation, staining, and imaging.

Consequently, to develop our throughput against SARS-CoV-2 we developed a luminescence cell-based assay using the highly permissive A549 cell line expressing both ACE2 and TMPRSS2 and the ic-SARS-CoV-2-nLuc reporter virus for establishing this drug screening platform. In conclusion, our semi-automated drug-screening platform offers a rapid, efficient, and scalable solution for identifying potential antiviral compounds against SARS-CoV-2. By utilizing a nano-luciferase reporter virus, this platform bypasses time-consuming virus inactivation and staining steps, enabling accelerated screening in both low- and high-throughput formats. The successful identification of 48 potential antiviral hits from the MMV Global Health Library and the confirmation of3 promising compounds underscores the platform’s reliability and versatility. This platform technology and validation protocol can be adapted for future use against other viruses, offering a valuable tool for global health and pandemic preparedness

## Supplementary Material

1

Supplementary material associated with this article can be found, in the online version, at doi:10.1016/j.slasd.2025.100211.
